# Can a population study assess the impact of fetal monitoring technology?

**DOI:** 10.1111/aogs.14426

**Published:** 2022-07-25

**Authors:** Jørg Kessler, Torbjørn Eggebø, Anne Flem Jacobsen, Trond M. Michelsen, Svein Rasmussen, Branka M. Yli

**Affiliations:** ^1^ Department of Obstetrics and Gynecology Haukeland University Hospital Bergen Norway; ^2^ Research Group for Pregnancy, Fetal Development and Birth, Department of Clinical Science University of Bergen Bergen Norway; ^3^ Department of Obstetrics and Gynecology Stavanger University Hospital Stavanger Norway; ^4^ Center for Fetal Medicine Trondheim University Hospital (St. Olav's Hospital) Trondheim Norway; ^5^ Department of Clinical and Molecular Medicine Norwegian University of Science and Technology Trondheim Norway; ^6^ Department of Obstetrics and Gynecology Oslo University Hospital Oslo Norway; ^7^ Institute of Clinical Medicine University of Oslo Oslo Norway

Sir,

In our opinion, the study by Blix et al. “The impact of the introduction of intrapartum fetal ECG ST segment analysis. A population study”^1^ has substantial weaknesses that should have been addressed in the article. We believe a more balanced presentation and discussion is needed.

For obvious reasons *intra*partum fetal monitoring cannot prevent *ante*partum stillbirth. The latter accounts for more than 90% of all stillbirths, and those are irrelevant as an outcome measure in the context of this study. Although the categorization into intrapartum and antepartum stillbirths was available from the Medical Birth Registry of Norway, the authors seem to have analyzed stillbirth as a total.

The authors rejected the possibility of a type‐2‐error based on the large sample size. During the study period, the total number of *intra*partum stillbirths in Norway decreased significantly from around 1/1000 to 1 to 2/10 000 (K. Laine, pers. comm., Figure 1). These numbers are very low, and we question whether the study has sufficient power to investigate hospital and time different effects of the introduction of the ST analysis (STAN) technology on intrapartum mortality.

**FIGURE 1:**
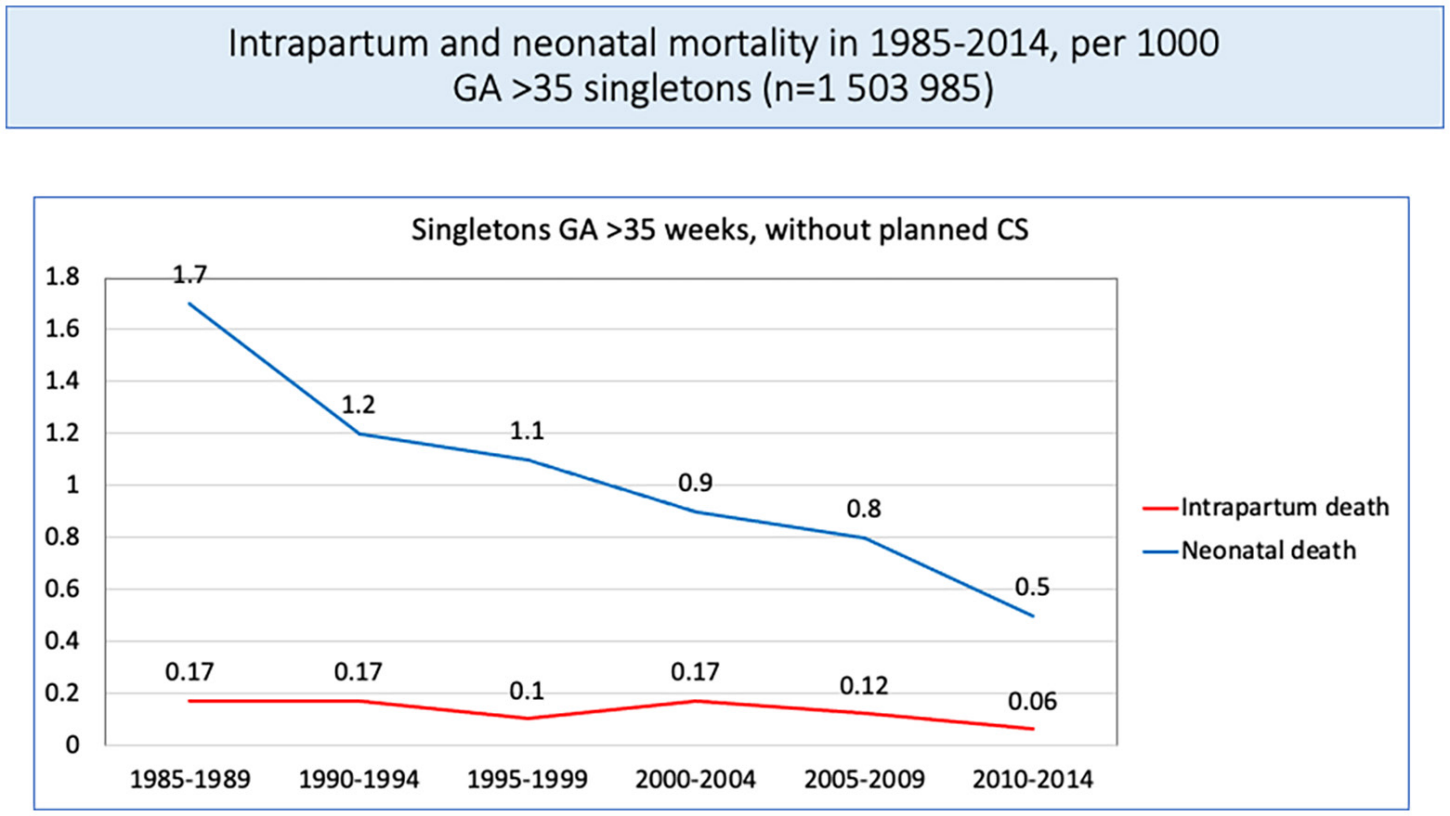
Intrapartum and neonatal mortality in Norway, 1985–2014, singletons, >35 weeks, planned cesarean section excluded. CS, cesarean section; GA, gestational age

Further, intrapartum monitoring cannot prevent neonatal death related to congenital malformations accounting for 30%–40% of all neonatal deaths at term.^2^ Thus, the number of theoretically preventable neonatal deaths is substantially lower than its total count. Unfortunately, this issue was not discussed by the authors.

Importantly, even at hospitals using STAN as an adjunctive technology, more than 60% of the deliveries had monitoring other than STAN (cardiotocography or intermittent auscultation).^3^ The *actual* method of intrapartum monitoring for *any* specific case in the study was not known, another major limitation not acknowledged by the authors. It is well known that use of historical controls and lack of individual data may lead to loss of information and biased conclusions.

The paper provides little data on fetal, neonatal or maternal outcome variables, and confounding factors (maternal risk factors) including changes in their prevalence over time. We also miss abovementioned data for the units *not* using STAN, accounting for 28% of all deliveries.

STAN is an adjunctive technology for *intra*partum fetal monitoring and cases delivered by cesarean section *before* the start of labor should be excluded. The authors apparently missed the exclusion of emergency cesarean section performed before the onset of labor. Those deliveries constitute a small number but carry a high risk of adverse outcome due to asphyxia, *not* eligible for STAN monitoring. Thus, the selection of the study population did not correspond to the population eligible for intrapartum monitoring.

Until the year 2000, the use of the STAN technology was rather sporadic—it was not based on an automated analysis of the fetal electrocardiogram, lacked robust clinical guidelines, and education and user certification were not prerequisites. The gradual development of technology and guidelines and their possible influence on outcome measures was not addressed.

In our opinion, the data presented in the study do not support its key message. The decreasing prevalence of birth‐associated cerebral palsy^4^ and intrapartum mortality in Norway (K. Laine, pers. comm., Figure 1), despite increasing maternal morbidity, deserves an explanation.

## References

[1] Blix E , Eskild A , Skau I , Grytten J . The impact of the introduction of intrapartum fetal ECG ST segment analysis. A population study. Acta Obstet Gynecol Scand. 2022;101:809‐818. 35288935 10.1111/aogs.14347 PMC9564625

[2] Blondel B , Eb M , Matet N , Breart G , Jougla E . La mortalité néonatale en France: bilan et apport du certificat de décès néonatal [Neonatal mortality in France: usefulness of a neonatal death certificate].In French. Arch Pediatr. 2005;12:1448‐1455. 16023843 10.1016/j.arcped.2005.05.009

[3] Kessler J , Moster D , Albrechtsen S . Intrapartum monitoring of high‐risk deliveries with ST analysis of the fetal electrocardiogram: an observational study of 6010 deliveries. Acta Obstet Gynecol Scand. 2013;92:75‐84. 22897758 10.1111/j.1600-0412.2012.01528.x

[4] Hollung SJ , Vik T , Lydersen S , Bakken IJ , Andersen GL . Decreasing prevalence and severity of cerebral palsy in Norway among children born 1999 to 2010 concomitant with improvements in perinatal health. Eur J Paediatr Neurol. 2018;22:814‐821. 29779984 10.1016/j.ejpn.2018.05.001

